# Lead pollution in soils within the African mining landscapes: current status, impacts, and nature-based interventions

**DOI:** 10.1007/s10661-026-15492-x

**Published:** 2026-05-27

**Authors:** Mutinta Malawo Matungu, Phenny Mwaanga, Theodore Mulembo Mwamba, Lee Mudenda, Stephen Syampungani

**Affiliations:** 1 Chair of Environment and Development, Oliver R. Tambo Africa Research Chairs Initiative (ORTARChI), Copperbelt University, 21692, Kitwe, Zambia; 2 Department of Zoology and Aquatic Sciences, School of Natural Resources, Copperbelt University, Jambo Drive, Riverside, 21692, Kitwe, Zambia; 3 Department of Environmental Engineering, School of Mines and Mineral Sciences, Copperbelt University, 21692, Kitwe, Zambia; 4 Faculty of Agriculture and Environmental Sciences, University of Lubumbashi, 1825, Lubumbashi, Democratic Republic of the Congo; 5 Department of Mines, School of Mines and Mineral Sciences, Copperbelt University, 21692, Kitwe, Zambia; 6 Forest Science Postgraduate Program, Department of Plant and Soil Sciences, Plant Sciences Complex, University of Pretoria, Private Bag x20, Hatfield, Pretoria, 0002 South Africa

**Keywords:** Food chain transfer, Human health risk, Lead pollution, Mitigation strategies, Nature-based solutions

## Abstract

Lead (Pb) pollution is a persistent environmental and public health crisis worsened by mining activities in African mining landscapes. Pb is a non-biodegradable toxicant that can enter the food chain via surface deposition or plant uptake from the rhizosphere. This review synthesizes the current status of Pb pollution, the food chain as a pathway for exposure, associated health challenges, and intervention strategies for Pb contamination in African mining areas. Following PRISMA guidelines, 92 peer-reviewed articles from 2000 to 2024 were identified across multiple databases. Original articles from reputable journals with complete metadata, abstracts, and full texts were included, while duplicates, theses, non-English studies, and articles outside this scope were excluded. Results show that soil Pb levels in West and Southern Africa exceeded permissible limits, while monitoring gaps exist in Northern and East Africa. The review notes Pb bioaccumulation in food crops, posing major health risks, especially to children, necessitating long-term interventions. Although bioremediation and amendment-assisted phytoremediation have shown promise, their suitability for large-scale application and community replicability requires further study. Amendment-assisted phytoremediation with biochar has shown potential to stabilize Pb in soil, but its effectiveness depends on many variables and requires adapting methods to local conditions. There is a significant knowledge gap regarding the long-term stability of sequestered Pb and the replicability of interventions across varying environments. Future research should focus on optimizing local, cost-effective remediation and establishing unified monitoring frameworks to protect food security and public health continent-wide.

## Introduction

The mining industry contributes approximately 45% to the global economy (Global investor Commission on Mining 2030, 2024), significantly strengthening economic growth by supporting sectors such as construction and manufacturing (Signé & Johnson, 2021). Africa is among regions where the mining industry is particularly flourishing, with abundant mineral resources, including a vast reserve of lead (Pb) that accounts for a significant share of global resources (International Lead and Zinc Study Group (ILZSG), 2023). Pb is a non-biodegradable element and is typically associated with minerals such as sulphides (galena, PbS, and anglesite, PbSO_4_), oxides, and carbonates (cerussite, PbCO_3_) (Hirwa, 2019). The most prevalent form of Pb in Africa is Galena or lead sulphide (PbS), usually occurring in association with sphalerite, which is the main Zn ore, while anglesite (PbSO) and cerussite (PbCO_3_) are secondary minerals occurring as a result of weathering of Galena (Baieta et al., 2021; Boussen et al., 2010; Doumo et al., 2022; Elom, 2018; Mondillo et al., 2018; Snodgrass, 1986). With the rising demand for base metals in recent years, Africa’s role in the Pb supply chain is expected to expand (ILZSG, 2023). The major Pb producers on the continent include Nigeria, Burkina Faso, Morocco, Namibia, and South Africa, collectively accounting for approximately 2% of global output in 2022 (0.1 million tons) (ILZSG, 2023). The strategic importance of Pb should not be understated. This metal stands out as one of the most versatile because it has a wide range of applications, including battery production, shot and ammunition, cable sheathing, Pb compounds, and other alloys (Boldyrev, 2018).

Despite the advantages associated with Pb mining, a considerable amount of non-biodegradable and non-essential Pb has been released into the environment (Tirima et al., 2015). While both organic and inorganic forms of Pb are released into the ambient environment, organic Pb compounds exhibit higher toxicity to biotic entities via the food chain, whereas inorganic forms are primarily found in soil, dust particles, and old paint. Human exposure to environmental Pb can occur through ingestion and inhalation of Pb-contaminated dust particles, dermal contact and geophagia (Anka et al., 2020; Iavazzo et al., 2012; Kříbek et al., 2015; Naicker et al., 2013). Consequently, mine workers and communities living near mining operations and allied industries face high risks of long-term exposure to Pb-contaminated dust particles (Ajayi et al., 2014; Mwaanga et al., 2019). In agricultural landscapes adjacent to mines or mineral processing plants, Pb contamination can also permeate agricultural and garden soils, entering the food chain. Pb in the food chain, insidiously accumulates in plants, animals and ultimately humans (Mwelwa et al., 2023; Tirima et al., 2017; Yoshii et al., 2020).

Pb toxicity in humans is commonly assessed by blood lead levels (BLL). A BLL exceeding 5 μg/dL is considered a reference value, while levels ≥ 45 µg/dL require initiation of chelation therapy (Olusegun & Schrauzer, 2010). The toxic effects of Pb can adversely affect the neurological, cardiovascular, renal, reproductive, and hematopoietic systems, with the neurological and hematopoietic systems being the most frequently and severely affected (Bose-O’Reilly et al., 2018; Cobbina et al., 2015). In 2021, Pb exposure was linked to over 1.5 million deaths globally, predominantly due to cardiovascular issues (Institute for Health Metrics & Evaluation, 2024). Between 2009 and 2010, Nigeria experienced a tragic Pb pandemic that claimed the lives of over 100 children below the age of 5 years old (Lo et al., 2012). In Kabwe, Zambia, a study by Yabe et al. (2020) reported that approximately 79% of 562 sampled children had BLLs greater than 5 µg/dL, and 23% had BLLs exceeding 45 µg/dL, indicating the need for chelation therapy.

While chelation therapy stands out as a treatment for Pb poisoning (Olusegun & Schrauzer, 2010), like most diseases, prevention of Pb poisoning through environmental remediation is key. However, historical and ongoing uses of Pb in products, such as automobile batteries, paint pigments, solder, gasoline additives, pipes, and pottery glaze, contribute to persistent exploitation and elevated exposure risks for humans. This sustained exposure is particularly pronounced in developing regions, such as Africa (Bonnifield & Todd, 2024), a concern recognized for many years (Nriagu et al., 1996a, 1996b).

Growing concerns about Pb pollution have led to various efforts to reduce human exposure, including behavioural modifications (Tirima et al., 2016), in situ management of Pb (Kříbek et al., 2019), and removal of Pb from contaminated environments (Tirima et al., 2015). Although substantial data exist on Pb pollution in Africa, they are often scattered. Early literature reviews, dating back to 1986, focused on pathways such as dust inhalation (Nriagu, Jinabhai, et al., 1996; Snodgrass, 1986), consumption of contaminated baby food, water, traditional medicines, and cosmetics, and mother-to-child Pb transfer (Nriagu, Jinabhai, et al., 1996), as well as the health-related effects of childhood BLLs (Chukwuma & Chukwuma Sr, 1997). However, these studies are outdated and may not accurately reflect the current status of Pb pollution in Africa, particularly regarding the potential role of the food chain as a pathway for Pb contamination, which has often been overlooked.

More recent reviews within the African context have highlighted soil Pb accumulation levels (Burga & Saunders, 2019; Lombe et al., 2021), health effects (Nakata et al., 2022), and remediation approaches (Burga & Saunders, 2019; Lombe et al., 2021; Nakata et al., 2022). However, these reviews are also largely silent on the food chain as a significant pathway for pollution. Furthermore, these studies primarily provided country-specific information on Pb pollution, such as for Zambia. Other country-specific reviews, focusing exclusively on Nigeria, are reported by Yabe et al. (2010), who examined Pb pollution from gasoline and the poor battery recycling, while Obeng-Gyasi (2019) highlighted issues with electronic waste recycling and the consumption of canned food, and Nkwunonwo et al. (2020) summarized literature on the vulnerability of Nigeria’s food chain to heavy metal pollution, thus emphasizing the need for caution regarding consuming certain foods. The review by Nkwunonwo et al. (2020) is an exception because it investigated the food chain as a non-negligible pollution pathway in Nigeria.

As can be noticed, despite extensive documentation of lead (Pb) levels in mining soils across Africa, a significant research gap persists in getting a consolidated view linking the status of soil Pb contamination to Pb dynamics in the soil–plant–environment system as pathways of pollution, which is needed to develop practical, scalable remediation initiatives. This review aims to present an updated, comprehensive analysis of Pb pollution and associated remediation strategies in the African context, integrating recent developments across the continent. The specific objectives of this study include: (i) synthesizing current data on soil Pb concentrations, sources, and contamination pathways in African mining regions, while evaluating the extent of Pb transfer into the food chain within these landscapes; (ii) assessing the potential human health risks posed by Pb-contaminated soils and the consumption of affected crop products in mining-impacted communities; and (iii) evaluating the effectiveness of existing soil remediation strategies and amendments in mitigating Pb bioavailability and plant uptake in the context of African mining landscapes. 

## Methodology

### Scope of literature search

This study employed a systematic review approach, adhering to the Preferred Reporting Items for Systematic Reviews and Meta-Analyses (PRISMA) guidelines and checklist (Page et al., 2021), to ensure scientific rigour, transparency, and replicability. Inclusion criteria comprised original research articles published between 2000 and 2024 in reputable journals with complete metadata, abstracts, and full texts. We also conducted a citation search on the documents retrieved to identify additional relevant studies, and then a purposive search of related research and review articles was done on Pb pollution reported outside Africa for comparative purposes. This comprehensive search used four reputable electronic databases i.e. Science Direct, PubMed, Google Scholar, and Semantic Scholar. Search terms included the following: “lead contamination”; “African mining soils”; “food chain transfer”; “human health risk”; “bioremediation”; “phytoremediation”; and “biochar”. The study excluded (i) articles published in journals that are not peer-reviewed, (ii) duplicate articles, (iv) theses, (iv) studies not published in English, and (v) studies outside the scope of this study.

To minimize bias, the Authors independently used different synonyms of key research terms, initially extracting all articles that fit the search criteria. The extracted articles were then imported into Mendeley for deduplication and screening. In Mendeley, the Authors verified article metadata for completeness, ensuring that each article included the journal name, author(s), title, year of publication (and month), abstract, page numbers or article number, keywords, volume and DOI (if available). Articles that did not fit this criterion were excluded from the study. Thereafter, the authors conducted a title and abstract screening, during which more studies were excluded being outside the scope of the study. Full-text articles were then downloaded and summarized in Microsoft Excel under different themes that included the extent of soil Pb contamination in the African mining landscapes, effects of Pb pollution on public health in African mining areas, bioremediation of Pb-polluted soil in African mining landscapes, and phytoremediation of Pb-polluted soil in African mining landscapes. Possible limitations of this systematic review include the restriction to studies published in English, the time frame (2000–2024), and the exclusion of grey literature not indexed in major databases.

### Articles retrieved

**Fig. 1:**
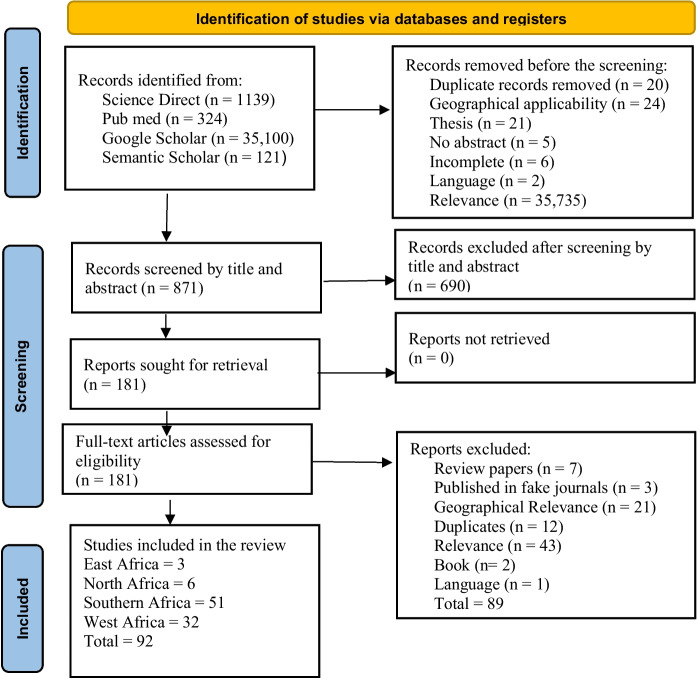
PRISMA flow diagram depicting the method used in the selection, screening, and inclusion of articles and related outcomes in the systematic review

Identification, screening and selection of reviewed research articles followed the PRISMA flow diagram (Fig. 1). Initially, 36,684 studies were identified from the different databases. Of these, 35,813 were removed in the initial screening based on geographical and study relevance. The remaining 871 articles were screened using the title and abstract fields in Mendeley, resulting in 690 exclusions. Out of 181 articles sought for retrieval, 89 were excluded after full-text screening. Ultimately, a final set of 92 studies was included and processed according to the PRISMA workflow (Fig. 1).

## Bibliometric analysis

**Fig. 2:**
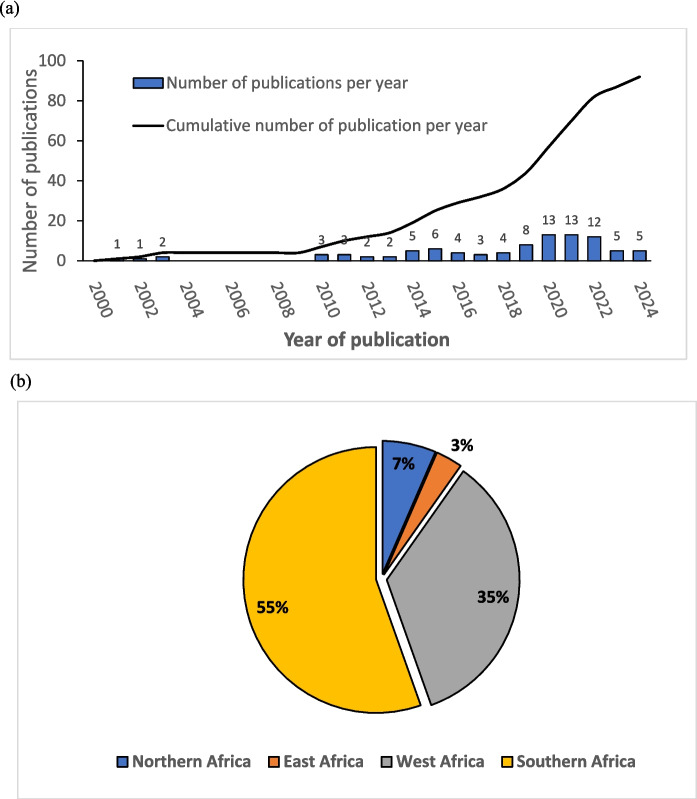
Results of bibliographic analysis: **a** Annual and cumulative number of publications, **b** Regional proportion of reviewed publications

Our bibliometric analysis provides the trend of information regarding the number of Pb-related publications per year (Fig. 2a), and the distribution of reviewed papers per sub-regions in sub-Saharan Africa (Fig. 2b). Based on results from Fig. 2a, there is an increase in studies conducted on Pb pollution in the African context, with a steady increase in published studies from 2010 onwards. Notably, a patent increase in the number of publications is noted from 2019. A very limited number of studies were recorded before 2010, with virtually no publications observed for the years 2004–2009. The growing interest in Pb pollution studies from 2010 coincides with the tragic 2010 Pb catastrophe in Nigeria that resulted in severe health challenges and fatalities that might have further raised public awareness of Pb health threats on the continent. Data on the sub-regional redistribution of the explored publications revealed that a larger proportion of Pb-related studies in Africa have been carried out in Southern Africa (55%), followed by West Africa (35%), whereas Eastern and Northern Africa have limited studies on Pb pollution, with respectively 7% and 3% of the reviewed papers (Fig. 2b).

## Soil Pb contamination and associated environmental impacts in African mining landscapes

### Soil Pb concentration and related sources of contamination

Across Africa, Pb pollution often indicates the broader environmental burden linked to the mining and processing of Pb-Zn ores, gold (Ag) ores associated with Pb, and to a lesser extent, copper (Cu), cobalt (Co), and tungsten. The extraction and processing of primary Pb and Zn ores, often in polymetallic deposits, constitute an important source of environmental Pb contamination in Africa. Interestingly, because Pb is non-biodegradable, it can persist in the environment for years after extraction and deposition. This is evident from the consistently elevated Pb concentrations reported in Kabwe, where Zn/Pb mining operations officially ceased in 1994, yet Pb concentrations remained elevated for multiple decades, demonstrating the enduring effects of Pb-Zn mining and processing activities (Table 1). For example, Chukwuma & Chukwuma (1997) reported high Pb levels in residential soils in Kasanda Township, approximately 2 km northwest of the smelter site, with up to 2400 mg kg^−1^ total Pb. These observations are consistent with those of Caravanos et al. (2014) and Kříbek et al. (2019), whose studies in residential areas surrounding the Kabwe lead/zinc mine reported Pb concentrations in soil as high as 40,692 mg kg^−1^ Pb (Caravanos et al., 2014; Kříbek et al., 2019). Similarly, in Touiref, north-west Tunisia, soil samples collected from the mouth of the Oued Sidi Bou Said river (draining Pb tailings deposited from the Touiref Pb/Zn mine) showed contamination levels up to 7230 mg kg−1 in the top 10 cm of soil thus highlighting the impact of primary Pb/Zn mining activities (Othmani et al., 2015).

**Table 1:** Regional Pb contamination levels and regulatory thresholds in African studies

Country	Region	Mineral mined	Agri soil (mg kg^−1^)	MPL (mg kg^−1^)	RESI-soil (mg kg^−1^)	MPL (mg kg^−1^)	CONT-soil (mg kg^−1^)	MPL (mg kg^−1^)	Reference
Tunisia	Fedj Lahdoum	Pb-Zn mining	3 646	100 (CCME, 1991)		300 (CCME, 1991)			Babbou-Abdelmalek et al. (2011)
Zambia	Kabwe	Pb-Zn mining					4900–42,200	n/s	Leteinturier et al. (2001)
	Kabwe						9.0 759	35 (WHO)	Tembo et al. (2006)
	Kasanda, Kabwe				3008	120 (USEPA, 2003, 2004)			Nakayama et al. (2011)
	Makandanyama, Kabwe				1613				
	Chowa				1233				
	Mutwe Wansofu, Kabwe				1148				
	Makululu, Kabwe				870				
	Luangwa, Kabwe				507				
	4 km^2^ mine, Kabwe				1470			400 (CCME, 1991)	Caravanos et al. (2014)
	0–5 km zones from the Kabwe mine				20–10,000	n/s			(Uchida et al., 2017)
	Kabwe town			70 (CCME, 2007)		140 (CCME, 2007)	40,692		Kříbek et al. (2019)
	Kabwe mine vicinity and a remote area					35 (WHO) and 140 (CCME, 2007)	16,000		Baieta et al. (2021)
	community playgrounds in Kabwe				265–3320	37 background Pb soil			Nakamura et al. (2021)
Namibia	Tsumeb, Oshikoto Region	Pb-Zn mining					111–8170	-	Mihaljevič et al. (2015)
Nigeria	Abare	Au mining and processing					1266	300 (EU; UK), 150 (USA) and 70 (CS	Abdu and Yusuf (2013)
	Enyigba State						1116.8 ± 43.2	420 (USEPA, 1993)	Oti, (2015)
	Anka		2246.55	300					Mohammed and Abdu (2014)
	Alibaruhu		2314.15	70 (CCME, 1991) and 100 (UK)					Obiora et al. (2016)
	Ishiagu-Enyigba		1634.44						
	Ameka		132.4						
	Zamfara Residential compound		1029.42	400 (USEPA)			1523.99	400 (USEPA)	Udiba et al., (2019)
	market square Zamfara						1404.57		
	old grinding mills Zamfara						6724.68		
	new grinding mills Zamfara						68.79		
	Anka				1322.5	100 (FAO, 1999; WHO,1999)			Akpanowo et al. (2020)
	Abare						354.8	400 (USEPA, 2004)	Darma et al. (2022)
	Dareta						72.09		
	Bagega						29.94		
Cameroon	Bétaré-Oya	Au mining					24.2	70 (Canadian standard)	Doumo et al. (2022)
Rwanda	Burera District	Tungsten (W), Pb, tin, etc. miming					52	25 g	Hirwa (2019)
CONGO DR	Kashamata	Cu and Co mining			1427	100 (WHO)		400 (CCME)	Katebe et al. (2024)
	Manoah Kinsevere				2472				
	Chem-chem				1255				
Kenya	Migori gold belt	Au mining					5.5–619	70 (FAO,1991; WHO, 1999)	Ngure et al. (2014)
	Southwest region						244.4	n/s	Odumo et al. (2018)
	Migori gold belt						15.5–705.9	10 (FAO, 1985; WHO, 1999; GSR)	Ngure & Kinuthia (2020)
South Africa	Bertrams	Au mining			1070.55	230 (SA) 120 (CCME)			Mathee et al. (2018)
	Riverlea				442.65				
	Bramfischerville				146.05				
	Hospital Hill				121.6				
	Ekuhurleni, Gauteng Province, South Africa		20–123.7	40 (USEPA)					Okereafor et al. (2019)

*AGRI* agriculture, *RESI* residential, *CON* contaminated soil with unspecified land use, *MP*L maximum permissible limit; *USEPA* United States Environmental Protection Agency; *CCME* Canadian Council of Ministers of the Environment guidelines; *CS* Canadian Standards; *FAO* Food and Agriculture Organization; *WHO* World Health Organization; *GSR* German Soil Regulation; *EU* European Union permissible limit; *UK* United kingdom; *USA* United States of America; *SA* South African reference value; *n/s* not specified

Beyond the extraction of lead and zinc ores, gold mining and related processing activities are a significant source of Pb contamination, particularly in the artisanal and small-scale mining (ASM) sector. For example, in Kenya, Pb concentrations in soil samples collected in areas surrounding rivers and mines have been reported to exceed the maximum allowable concentration (Ngure et al., 2014; Odumo et al., 2018; Table 1) in the Migori gold belt and Transmara. Similarly, in South Africa, the four suburban residential areas of Johannesburg, close to a gold mine and associated mine wastelands, showed a high Pb concentration exceeding both the South African and Canadian reference values for contaminated soil (230 mg kg^−1^ and 120 mg kg^−1^, respectively) (Mathee et al., 2018). In Anka, North-West Nigeria, artisanal gold mining and processing sites were observed to have Pb concentrations exceeding the WHO/FAO (1999) permissible limit in both soils and sediments (Akpanowo et al., 2020). Conversely, Cameroon-sampled soil sediments associated with gold mining showed an average Pb concentration of 24.2 mg kg^−1^, which, although higher than the upper continental crust reference value of 14 mg kg^−1^, was lower than the Canadian soil quality standard of 70 mg kg^−1^ (Doumo et al., 2022). These findings suggest that gold ore deposits are associated with other minerals, including Pb, and can lead to significant Pb deposition in the ambient environment. Such Pb deposition in residential areas is a cause for concern in African mining areas due to the risk of direct or indirect ingestion of Pb-contaminated dust particles. While lead, zinc, and gold mining account for a substantial portion of the reported sources of Pb contamination, other mineral extraction activities, though less extensively documented in some regions, are increasingly recognized as significant contributors to elevated soil Pb levels in Africa. In this regard, copper mining activities have been recognized as a source of Pb pollution in Namibia, where Pb concentrations evaluated in soils within proximity to Tsumeb Pb and Cu smelter facilities (Mihaljevič et al., 2015) were observed to be high (8,174 mg kg^−1^), thus posing both an ecological and a health risk in residential areas (Mihaljevič et al., 2015). Like the situation in Namibia, Cu-Co mining has also been identified as an important source of soil Pb contamination in DR Congo (Katebe et al., 2024).

Burera District in Rwanda presents a distinct scenario of soil Pb contamination, which is associated with tungsten mining in the tungsten belt, comprising silica-clastic rocks with composition ranging from black shales to quartz-phyllites to quartzites (Hirwa, 2019). Hence, soil Pb levels around the Gifuwe mining sites were reported to exceed the permissible limit of 25 mg kg^−1^ under the German Soil Regulations (1999) (Hirwa, 2019). Further, quarrying activities have resulted in approximately 12 Mt of tailings being abandoned along the banks of the Moulouya River in Zeida, Morocco, leading to soil Pb levels ranging from 32 to 832 mg kg^−1^ (Bouzekri et al., 2019), underscoring the distinct mining-industry origins of Pb contamination.

Recognizing the environmental risks associated with metal contamination, international and national environmental regulatory bodies have established permissible soil Pb standards to protect environmental integrity and public health. However, as shown in Table 1, most African countries do not have a specific maximum allowable Pb level in soil. In most cases, international standards for International regulatory bodies such as World Health Organization (WHO), Food and Agriculture Organization (FAO), United States Environmental Protection Agency (USEPA), European Union permissible limit (EU), Canadian Council of Ministers for the Environment(CCME), Canadian Standards (CS), and German Soil Regulation (GSR) have been applied in the regulation of soil Pb contamination. Among these regulatory standards, CCME, USEPA and FAO/WHO standards appear to be the most commonly used. A notable exception is, however, provided by Mathee et al. (2018), who utilized a South African reference for both Pb and Arsenic (As) contamination in residential garden soil, although the Canadian Pb and As reference levels (120 mg kg^−1^ and 18 mg kg^−1^, respectively) are more protective than the South African reference levels (230 mg kg^−1^ and 48 mg kg^−1^, respectively). Overall, the regulatory void observed in various Pb pollution studies across the African continent (Table 1), coupled with the continent’s rich and diverse mineral endowments (Signé & Johnson, 2021), poses a challenge, as mining activities significantly contribute to soil Pb concentrations and pollution. Pb/Zn mining and Au mining stand out as the most prominent causes of Pb pollution in African mining landscapes (Table 1). Further, while Pb contamination has been reported in Southern and West Africa, a significant monitoring gap is evident in Northern and Eastern Africa where limited studies have reported Pb contamination in soil (Babbou-Abdelmalek et al., 2011; Ngure & Kinuthia, 2020; Ngure et al., 2014; Odumo et al., 2018; Table 1).

## Pathways of Pb contamination and Pb level in crop products in African mining regions

The diverse sources of Pb contamination from mining activities and the widespread soil Pb contamination across Africa inevitably expose populations to health risks. Pb contamination in African mining landscapes is driven by dust emitted during mineral processing and by the erosion of mine and ore-processing waste into the atmosphere and ambient environment. The lack of adequate tailings decommissioning has left tailings and waste rock that serve as a continuous source of Pb contamination. In the environment, deposited Pb interacts with native minerals, often resulting in Pb contamination that exceeds acceptable thresholds (Mandal, 2022; Yahaya et al., 2022).

### Atmospheric Pb particles in African mining landscapes

Atmospheric lead (Pb) typically occurs as fine particulate matter that integrates into airborne soil dust, facilitating inhalation by human and animal populations within a contaminant’s catchment area (Nicholas & Fuller, 2020). The dispersion of these particles is primarily governed by wind speed and direction, creating immediate pollution pathways that show pronounced seasonal variability. For instance, simulation models indicate that lead dispersion from mining sites is strongly influenced by wind during the dry season, whereas the effect is negligible during the rainy season (Nakamura et al., 2021). This pattern is evident in Kabwe, where prevailing easterly and south-easterly winds have created contamination hotspots in the western and southern districts, such as Kasanda and Makululu, while more distant areas, such as Mpima, remain relatively unaffected (Moonga et al., 2022). Therefore, given the spatiotemporal variability of wind-driven transport, establishing a definitive link between contamination and its source requires the use of lead isotopic signatures as a forensic tool (Monna et al., 2006).

To accurately differentiate between natural and anthropogenic lead sources, researchers utilize lead isotopic signatures, which provide distinct fingerprints for specific emission groups despite the high operational costs involved (Monna et al., 2006). This isotopic approach is frequently applied to biological receptors such as trees, mosses, and lichens, which serve as integrated monitors of atmospheric quality. While atmospheric Pb research remains limited across the African continent, studies using mosses and lichens in Harare, Zimbabwe, and the Western Cape, South Africa, have demonstrated a clear correlation between Pb concentrations and proximity to the central business district (Mupa et al., 2013; Ndlovu et al., 2019). Similarly, in Nigeria, atmospheric Pb was reported as exceeding the permissible limit of 30 ppm set by WHO after assessing its content in epiphytic moss *Polytrichum juniperinum* and the tree bark of trees such as *Azardirachta indica* along the Abuja-Lokoja highway as a biomonitoring tool (Kakulu, 1993; Ochala et al., 2021). Complementing these biological datasets, studies on inanimate surfaces and human-mediated pathways reveal the broader scope of lead’s persistence in the African environment.

Other indicators of atmospheric Pb include surface films and roadside soil samples. In Kampala, Uganda, atmospheric deposition has been identified as the dominant pathway for lead contamination, reflected in high concentrations within surface films (Nabulo et al., 2006). Notably, lead particles may also be transported from the workplace to the home via the skin and clothing of workers in industries processing Pb, extending the risk of pollution to their families (Tong et al., 2000). However, reports of atmospheric Pb in Africa primarily attribute Pb pollution to industrial and vehicular emissions (before restrictions on the use of leaded gasoline were introduced) and to the use of leaded paints and pipes (Mupa et al., 2013; Nabulo et al., 2006; Tong et al., 2000). Therefore, a significant research gap exists with regard to atmospheric monitoring of airborne Pb particles in most Pb pollution-affected African mining areas.

### Pb-contaminated water as a pollution pathway

In the African mining landscape, hydrogeochemical mobilization of Pb into surface and groundwater poses a threat to water security. Atmospheric Pb is deposited on water and soil surfaces, where oxidative weathering of sulphide minerals can lower the pH of infiltrating water and runoff, thereby significantly increasing Pb solubility and mobility into local water bodies and aquifers (Siame et al., 2025). Similar to atmospheric Pb contamination, the existence of Pb in the aqueous phase is seasonal, resulting in peak metal loads in the dry season in stagnant water and shallow wells, which usually serve as primary drinking sources in peri-urban communities and vice versa in the rainy season (Aboagye & Zume, 2019). In the Kabwe Zn/Pb mining areas, water samples were reported to have Pb exceeding 0.010 mg/L, the WHO permissible limit (Siame et al., 2024). Pb-contaminated leachate may also originate from dump sites with leachate seepage like in Uganda, where water from Lake Victoria (the primary source of tap water in Kampala), was found to contain Pb levels exceeding the maximum WHO limit for lead in drinking water (10 µg/g) (Cusick et al., 2018; Graber et al., 2010; Makokha et al., 2008). These risks can further be observed in contaminated water from both the lake and taps in the vicinity of Lake Victoria basin around Kampala in Uganda, with lead levels at an average of 1.23 and 0.44 mg/100 ml (from the lake and the taps respectively). These figures are significantly higher than recommended levels of surface freshwater (0.01 mg/100 ml) and drinking water (0.001 mg/100 ml) attributed to anthropogenic activities (Akpanowo et al., 2021; Mghweno et al., 2008).

### Animals and animal products as pathways for Pb exposure

Edible animals such as cows, goats, insects, and chickens, and their products such as milk and eggs, have been highlighted as potential secondary Pb pollution pathways. The risk of toxicity stems from the gastric availability of Pb in the environment when ingested by animals. This process is governed by an interplay of biophysical and chemical factors, including pH-dependent dissolution, particle size, and the chemical matrix’s reactivity, with respect to in vivo mobilization (Hemphill et al., 1991; Mushak, 2015). Therefore, contamination may not be directly correlated with pollution due to the dynamics described above. In Kabwe, Zambia, free-range chickens scavenging in contaminated soils were found to have Pb levels in their liver, kidneys, and muscle that far exceeded international safety limits for human consumption (0.5 mg/kg and 0.1 mg/kg wet weight, respectively), whereas broilers raised on controlled feed remained significantly less affected (Yabe et al., 2013). Similarly, elevated Pb concentrations in fish from Lake Victoria highlight an aquatic pathway linked directly to water contamination (Makokha et al., 2008). Pb in edible insects collected in the Copperbelt province of Zambia was reported to be higher than the WHO permissible limit of 0.04. Furthermore, cattle blood in mining regions have shown high Pb loads (up to 90.6 µg/kg) and associated immunological alterations (Yabe et al., 2012). However, dietary risks from milk consumption have sometimes been found to fall within acceptable safety limits, suggesting that certain animal products may sequester lead differently than others (Yabe et al., 2012; Zyambo et al., 2022).

### Agricultural crops as a Pb pollution pathway

**Fig. 3:**
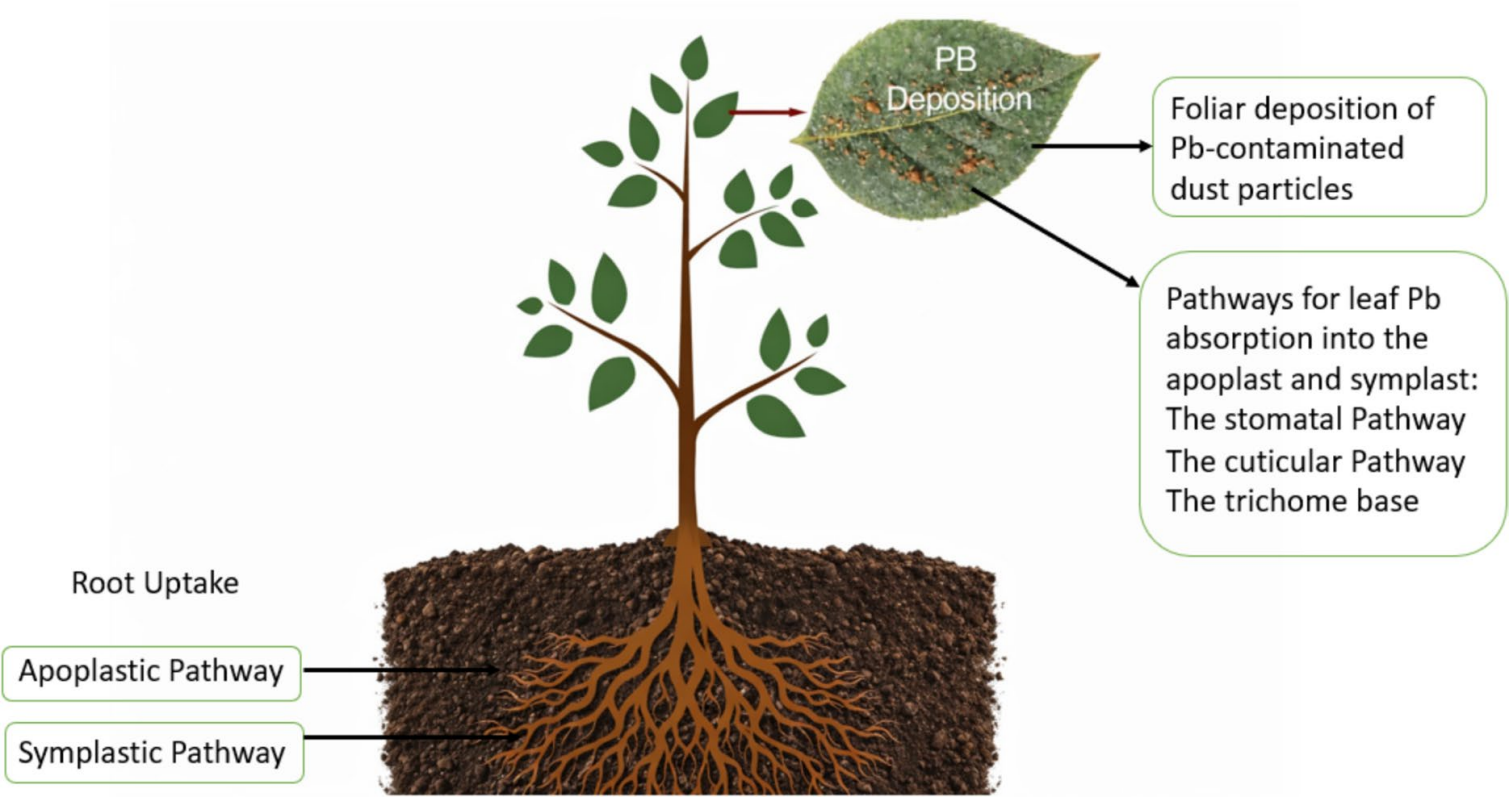
Plant Pb deposition and absorption through foliar and root pathways

While studies quantifying Pb pollution in the environment exist, the potential of the food chain as a pathway for pollution should not be underestimated. Agricultural land near mining areas poses a significant challenge to food security and human health due to the consumption of food crops grown in heavy-metal-contaminated soils. For example, agricultural soils sampled within the Lake Victoria basin in Kenya showed Pb concentrations exceeding both the FAO (1985) and WHO (1999) maximum allowable limits due to small-scale gold mining activities (Ngure & Kinuthia, 2020: Table 1). Similarly, studies of agricultural soils across West Africa have reported total Pb concentrations exceeding the 300 mg kg^−1^ threshold for agricultural soils and the maximum allowable limits set by US-EPA (1993), Canada (CCME, 1999) and Europe (EU, 2009) with Enyga community in Nigeria reporting the highest Pb in agricultural soil (2,246.55 mg kg ^−1^) (Table 1). These observations echo those from 1997, when Pb levels in soil in derelict Enyigba-Abakaliki, Nigeria, reached 23,000 mg kg^−1^ and up to 880 mg kg^−1^ in other parts of Abakaliki, reflecting Pb’s cumulative and non-biodegradable nature, and the legacy of mining activities (Chukwuma & Chukwuma Sr, 1997). Tunisia, DR Congo, and Rwanda also recorded Pb soil levels exceeding their country-specific maximum allowable limits in gardens and farmland (Babbou-Abdelmalek et al., 2011; Hirwa, 2019; Katebe et al., 2024; Table 1). However, because plant Pb accumulation does not always directly correlate with total soil Pb concentrations (Srivastava et al., 2022), understanding the contributions of both foliar deposition, leaf absorption and soil-plant Pb uptake through various pathways is essential for accurate risk assessment in agricultural settings (Kumar et al., 2020; Nkwunonwo et al., 2020; Shetty et al., 2025; Fig. 3). Plant roots absorb Pb from the soil through passive diffusion and facilitated transport through the symplastic and apoplastic pathways to different parts of the plants (Rahman et al., 2024). In plant leaves, deposited Pb particles can enter the plant through the stomata, cuticle, and trichome base and be transported to other parts of the plant through the phloem (Rahman et al., 2024).

Despite limited studies on atmospheric Pb monitoring and foliar absorption, the direct deposition of Pb-contaminated dust particles onto the aerial surfaces of crops has been reported to occur during plant cultivation and in post-harvest processing and transportation (Abdu & Yusuf, 2013). Depending on proximity to mines and mineral processing sites, Pb-containing airborne particles travel and settle on plant leaves, stems, and fruits. A general trend is evident in African studies, with reports indicating foliar Pb contamination during the plant growth period and during processing and transportation (Darma et al., 2022; Ngure & Kinuthia, 2020; Obiora et al., 2016; Orisakwe, Ebere, et al., 2017; Tirima et al., 2017). In Zamfara State (Nigeria) for example, pulverized crops were reported to have more than double the Pb contamination level of raw crop produce (Tirima et al., 2017). In a similar study, leaf and stem samples of cowpeas and sorghum showed lead concentrations up to 500 times the FAO/WHO (2001) recommended limit of 50 mg kg⁻^1^ for edible crops, attributed to dust from gold ore processing (Abdu & Yusuf, 2013). At the Associated Battery Manufacturing (ABM) plant in Nairobi, Kenya, Pb levels exceeding both the WHO and Kenyan acceptable limits (< 0.3 ppm) were reported in sampled plant leaf extracts, attributed to the absorption of Pb deposits on the plant leaf surface (Otieno et al., 2022). However, a monitoring gap remains in quantifying the contribution of foliar Pb uptake to the overall plant contamination and to the subsequent Pb pollution through the food chain in African mining landscapes.

In addition to foliar contamination, the soil itself is a sink for Pb, which crops can absorb in soluble forms through their root systems, leading to internal bioaccumulation. However, while total Pb deposited on soil surfaces increases the risk of contamination, the amount actually absorbed by plants depends on the bioavailable fraction of Pb in the soil (Kumar et al., 2020; Shetty et al., 2025). Once in soil, Pb strongly associates with clay minerals, Mn oxides, Al and Fe hydroxides, and organic matter, forming insoluble compounds such as Galena (PbS), lead (II) hydroxide [Pb (OH)_2_], Cerussite (PbCO_3_), and lead (II) phosphate [Pb (PO_4_)_2_] (Boldyrev, 2018). However, changes in the composition of these elements and other variables, such as soil mineralogy, microbial activity, ligand concentrations, and competing cations, may destabilize total Pb, thereby increasing its availability in more soluble forms, such as PbCO₃ (Awasthi et al., 2023; Nkwunonwo et al., 2020; Shetty et al., 2025). Once absorbed, Pb is generally poorly translocated from roots to shoots, but significant amounts can still reach edible parts. For instance, Oti (2015) and Obiora et al. (2016) reported that locally grown crops in the Enyigba community and Zamfara state had average Pb concentrations in roots, fruits, and vegetables exceeding the European Union (EU) (2006) and WHO/FAO (2007) permissible limits of 0.43 mg kg^−1^ and 0.3 mg kg^−1^, respectively (Table 2). Akpanowo et al. (2020) also reported Pb concentrations ranging from 12 mg kg^−1^ to 97 mg kg^−1^ in edible plants from artisanal mining communities in Anka, North-West Nigeria.

**Table 2:** Lead contamination in crops produced in African mining landscapes

*Country*	*City/state*	*Mineral mined*	*Food crop*	*Pb in Plant mg kg1*^*−1*^	*MAC mg kg*^*−1*^	*Reference*
***Nigeria***	Enyigba	Au mining	Potatoes	3.98	0.43 and 0.3 set by EU (2006) and WHO/FAO (2007)	Obiora et al. (2016)
			Yam	0.26		
			Cocoyam	0.54		
			Cassava	8.65		
			Potato leaves	3.53		
			Pumpkin leaves	5.46		
			casava leaves	3.61		
			lemon grass	137.83		
***Kenya***	Lake Victoria Basin	Au mining	Cabbage	0.17–176	0.3—FAO, 1985; WHO, 1999, and WHO/FAO. 2007	Ngure and Kinuthia (2020)
			Mango	0.3–7.5	0.3—EU 2002, FAO/WHO 2001, and FAO/WHO 1989	
			Potatoes	0.12–0.22	0.1—EU 2002 and FAO/WHO,	
			Maize	1.75–19.98	0.3—EU 2002 and FAO/WHO	
***Nigeria***	Enyigba	Pb/Zn mining	Okro	0.22	0.3 WHO	Oti (2015)
			Tomatoes	1.4		
			Pepper	1.84		
			African Paudauk	0.82		
			Red sandalwood	0.68		
			African Salad	1.42		
			Beni Seed Nauclea	3.71		
			African Peach	6.72		
			Lettuce	2.6		
			Guava	2.18		
			Mango	0.82		
	Abare	Au mining	Spinach	6.1–9.8	0.3 mg kg^−1^ FAO/WHO maximum allowable limits in vegetables (FAO/WHO 2001, 2007	Darma et al., (2022
	Bagega		Spinach	2.7–3.5		
	Dareta		Spinach	0.3–4.7		
	Abare		Maize	3.6 to 4.2	5 mg kg^−1^ FAO/WHO 2007	
	Bagega		Maize	1.9.1–2.5		
	Dareta		Maize	0.35–1.26		
	Begega farm	Au mining	Sorghum	0.93	n/s	Tirima et al. (2017)
			Maize	0.27		
			local rice	0.73		
			cow pea	0.24		
	Anka market		millet	0.53		
			maize	0.12		
			local rice	0.44		
			cow pea	0.36		
	Jos Plateau	Sundry mining (iron ore, tin, aluminum, etc.)	**Dilimi Rivers**		n/s	Orisakwe et al., (2017a, 2017b)
			Onions0.533			
			Green beans	0.646		
			Tomatoes	0.829		
			Cabbage	2.101		
			Lettuce	2.419		
			Spinach	0.953		
			Carrot	0.64		
			**Bukuru**			
			Carrot	1783		
			Cabbage	1460		
			Tomatoes	1889		
			**Burkin Ladi**			
			Red pepper	1,362		
			Brown Beans	0.510		
			Green beans	1723		
			Lettuce	0.910		
			Irish Potatoes	0.383		
			Onions	0.460		
	Jos Metropolis	*Tin mining*	*Annona senegalensis*	6.3	10	Mafulul et al. (2024)
			*Psidium guajava*	9.71		
			*Mangifera indica*	5.43		
			*Vernonia amygdalina*	12.53		
			*Vitex donniana*	13.79		

*FAO* Food and Agriculture Organization; *WHO* World Health Organization; *EU* European Union permissible limit; *n/s* not specified

Average Pb concentration in vegetables collected in the gold-mining state of Jos Metropolis, Nigeria was higher than the FAO/WHO (2001) maximum allowable concentration of Pb in vegetables (Orisakwe, et al., 2017a, 2017b; Table 2). In the Tin-mining area of Jos Metropolis, Nigeria, both medicinal and food crops were reported to have Pb levels exceeding the WHO maximum allowable concentrations (Mafulul et al., 2024; Orisakwe, et al., 2017a, 2017b; Table 2). Similar findings have been reported in Kenya, where Pb levels in various plants exceeded both the WHO and FAO contamination thresholds in the gold-mining area (Ngure & Kinuthia, 2020: Table 2). Unlike foliar contamination, which may be washed off, absorbed metals cannot be readily removed from the plant and therefore pose a persistent risk to the food chain. Overall, based on data gathered in this review (Table 2), plant leaves tend to have higher Pb concentrations than tubers and fruits. For instance, cassava leaves, pumpkin leaves, lettuce, and potato leaves were observed to have higher Pb concentrations than tubers such as yams, cassava and Irish potatoes cultivated in different parts of Nigeria (Ngure & Kinuthia, 2020; Obiora et al., 2016; Orisakwe, Ebere, et al. 2017: Table 2). Similarly, green leafy vegetables have been reported to have higher Pb concentrations than fruits such as mangos and guavas (Ngure & Kinuthia, 2020; Oti, 2015). This could be attributed to differences in the responses to lead exposure among these plant species.

In real-world agricultural settings within contaminated landscapes, both direct deposition and root uptake often coincide (Fig. 3), leading to a cumulative Pb burden in crops that significantly amplifies the overall health risk. Once settled or absorbed by the plant, given the probable tolerable daily intake (PTDI) of 0.0035 mg kg day^−1^ (JECFA 1999 and US EPA 2003), and the cumulative nature of Pb in the environment, it is necessary to implement mitigation measures that reduce or completely prevent Pb from entering the food chain. It is also evident from this review that most studies on Pb contamination of food chains have been conducted in West Africa, underscoring the need for further research to clarify the role of the food chain in Pb pollution within and around African mining landscapes. These findings highlight the urgent need for a thorough understanding of Pb transfer to crops grown in contaminated soils across sub-Saharan Africa, as well as the development of locally appropriate mitigation strategies. Social and economic profiles and dynamics of communities are also worth considering in this regard, as the dietary habits of local communities could influence their exposure to Pb pollution (Chukwu & Obiora, 2023; Yamada et al., 2023).

## Health risks associated with Pb pollution in African mining landscapes

The pervasive nature of Pb pollution, particularly in African mining landscapes, necessitates direct and effective approaches to accurately ascertain associated human health risks. The complex relationship between environmental Pb exposure and its internal absorption (Kříbek et al., 2019) can lead to severe health challenges. These challenges include long-term neurological impairment including blindness and deafness, anaemia, small stature and weakness, behavioural problems such as over mouthing activities, high child failure rate at school and convulsion-related deaths in children (Nakata, Hokuto, et al., 2021; Von Schirnding et al., 2003; Yabe et al., 2015; Table 3). However, while some studies have investigated and reported Pb-related health incidents, others have simply inferred potential health challenges based on known BLL correlations (Table 3). Biomonitoring plays a crucial role in assessing these risks. Generally, Blood lead level (BLL) is a widely used biomarker, with a benchmark of 5 µg/dL typically indicating a level above which adverse health effects are expected. In African mining landscapes, BLL assessment remains the most widely utilized bioindicator of Pb exposure, especially for diagnosing childhood Pb poisoning (Table 3). Other biomonitoring tools have also been proposed—such as bone and dental conditions for long-term exposure (Yamada et al., 2020), urine and feces reflecting absorbed and unabsorbed Pb (Yabe et al., 2018), or hair samples indicating long-term dietary Pb intake (Ngure & Kinuthia, 2020). However, only a few cases have been reported in which these alternative biomarkers have been used in African mining regions. These alternatives present their own challenges: bone samples reflect lifetime exposure, urine samples are difficult to obtain from infants, and feces primarily represent unabsorbed fractions, which may account for their limited use. Conversely, BLL represents the biologically active fraction of lead that is currently circulating and available to damage essential body organs. The availability of BLL, which correlates with acute adverse health effects, such as neurocognitive deficits in children or hypertension in adults, and the lack of standards for other biomarkers (such as urine or feces), make BLL a more reliable and relatable biomarker of Pb pollution (Gundacker et al., 2021; Sakai, 2000).

**Table 3:** Lead levels in children and adults across African mining landscapes

**Region**	**Country**	**Age range**		**Biomonitoring parameters**				**Health incident**	**Reference**
Southern Africa	Zambia, Kabwe		BLL (µg/dL)	Hair (mg kg^−1^)	Breast milk (μg/L)	Urine (Pb-USG)	Faecal (mg kg^−1^, dry weight)		
		2–8 years old	196 children were tested, and the mean BLL was 48.3, 26.5% exceeded the upper limit of 65.0						Caravanos et al. (2014)
		< 7 years old	18%, 57%, and 25% of children sampled in Chowa, Kasanda, and Makululu, respectively, had BLLs exceeding 65 µg/dL, with the maximum BLL being 427.8					Anaemia, small stature, and weakness	Yabe et al. (2015)
		< 7 years old	Makululu Kasanda			67.8 (GM) 12.1 (GM)	9.32 (Gm) 35.3 (Gm)		Yabe et al. (2018)
			Mean BLLs in infants and mothers were 18.0 and 11.3, respectively. over 70% mother and children had BLLs > 5		The overall mean concentration was 5.3 ± 7.0		Overall mean conc was 5.3 ± 7.0 and 39.2 ± 217.7	None of the infants and mothers had overt signs of Pb poisoning	Toyomaki et al. (2021)
		0–19 years	Children with BLL > 5 were estimated 1,190,789, while those with BLL > 10 were about 140,715						Nicholas and Fuller (2020)
		3 months to 9 years),	BLL ranged from 1.65 to 162 23% of the sampled children exceeded the BLL threshold of 45 mg/dL required for chelation therapy. A few children (5) exceeded 100 mg/Dl BLL						Yabe et al. (2020)
			The representative mean BLL was 11.9, and 74.9% of the residents had BLLs above 5						Yamada et al. (2020)
		0 to 15 years old	An average of 30.14 and a maximum of 162 among the 0–3 age group						Moonga et al. (2022)
		0–4 years	Average of 16.84 and in the range of 1.73–154.75						(Nakata, et al., 2021a, 2021b)
		5–17 years	19.63 and in the range of 1.62–66.89						
		> 18 female	11.63 and in the range of 0.79–57.87						
		> 18 male	Average of 13.23 and in the range of 0.95–63.63						
	Aggeneys, South Africa	Mean—8 years old	In Aggeneys and Pella respectively, 98% (range = 9.0–27.5 mg/dl) and 85% (range = 6.0–22.0 mg/dl) of children had blood lead levels > 10 mg/dl					Overactive children, abnormal mouthing activities and child failure rate at school	Von Schirnding et al. (2003)
	Johannesburg, South Africa	7–14 years and > 18 years old	Adult BLL ranged from 0.5 to 12.2, while in children, the range was 0.8 to16.1						Mathee et al. (2022)
	Mbare in Harare, Zimbabwe	± 3 years	The mean BLL was 4.3. 13.95% of participants had a BLL of > 5.0						Chagonda et al. (2023)
East Africa	Migori, Kenya			0.02–0.61 was within the MAC of 0.03 to 1.4 (EU, 2002, FAO/WHO, 1993, and FAO. 1985)					Ngure and Kinuthia (2020)
West Africa	Zamfara State, Nigeria	< 5 years of age	204 children were tested had BLLs ≥ 10, and 97% had BLLs ≥ 45					Vomiting, abdominal pain, headache, convulsions, long-term neurological impairment including blindness and deafness	Dooyema et al. (2012)
	Nasarawa state, Nigeria	Adults > 7 years of age	3.1 BLL with a range of 0.6–14.8 BLL					Child deaths	Bello et al. (2016)
		< 7 years of age	2.1 BLL ranging from untraceable to 9.5 BLL						
	Bagega community, Zamfara State, Nigeria	< 5 years	99.5% of the sample had elevated BLL > 10 BLL, and 91.4% had severe Pb poisoning of ≥ 45 BLL					Convulsion-related deaths in children	Ajumobi et al. (2014)
	Bagega, Nigeria	1–6 years	Approximately 400 to 500 children died of acute Pb poisoning associated with artisanal gold mining in Zamfara						Tirima et al. (2017)

*BLL* blood lead level, *GM* geometric mean, *MAC* maximum allowable concentration, *FAO* Food and Agriculture Organization, *WHO* World Health Organization

Children are the most vulnerable members of any society due to their hand-to-mouth tendencies, low body weight, sensitive cells, tissues, and organs (Akpanowo et al., 2021; Yabe et al., 2020; Yamada et al., 2020). A prominent case of Pb poisoning in children has been reported in Zambia, where children living in proximity to the old Kabwe Pb/Zn mine were diagnosed with BLL values > 10 µg/dL and, in some cases, exceeding 40 µg/dL (Table 3), a benchmark at which chelation therapy is needed (Bose-O’Reilly et al., 2018; Yabe et al., 2015). Reported symptoms in Kabwe, though from unpublished sources, included anaemia, intermittent abdominal pains, limb pains, memory problems, headaches, weakness in hands and feet, and seizures or convulsions (Bose-O’Reilly et al., 2018). In North-eastern Nigeria, 118 (25%) of 463 children under 5 years old were reported to have died of Pb poisoning due to the Pb exposure in artisanal gold mining communities from May 2009 to May 2010 (Dooyema et al., 2012). Among the 345 children under 5 who survived, 59% (204/345) were lead poisoned (≥ 10 µg/dL), with 97% (198/204) having BLLs ≥ 45 µg/dL, the threshold for chelation therapy (Dooyema et al., 2012). In Aggeneys (North-West Cape), a Pb mining town in South Africa, the average blood Pb levels were 16 mg/dL and 13 mg/dL in 1997, with similar results reporting in 2003 (Nriagu et al., 1997; Von Schirnding et al., 2003). South Africa (Johannesburg) and Zimbabwe (Harare) experienced cases in which children were most affected, although reported numbers are significantly lower than those in Zambia and Nigeria (Chagonda et al., 2023; Mathee et al., 2022; Table 3).

According to Nriagu et al. (1997), Africa is vulnerable to Pb pollution due to dusty environments, poor nutrition and the predominance of women and children in most communities. Further, the persistent industrial demand for Pb, as highlighted by Nicholas and Fuller (2020) and the World Health Organization (WHO) (2012), remains a significant environmental concern today. This underscores the urgent need for effective, adaptable and replicable Pb remediation strategies across Pb-contaminated African mining landscapes to mitigate further pollution and safeguard public health. Establishing sustainable remediation guidelines and strategies is paramount to preventing the exacerbation of contamination.

## Interventions and remediation of Pb-polluted landscapes

### Immediate interventions

Pb pollution in Africa’s mining landscapes poses immediate and long-term threats to both human and environmental health. While international and local authorities have suggested and implemented interventions in several African countries, the majority of reported efforts are concentrated in West Africa (Nigeria) and Southern Africa (Zambia) (Tables 4, 5, and 6). In Nigeria, various interventions have been implemented, including community awareness campaigns, excavation and replacement of polluted soils, relocation of ore processing activities away from villages, adoption of Pb dust-minimizing processing methods, and hygiene measures (Anka et al., 2020; Tirima et al., 2015). Hence, approximately 27,000 m^3^ of contaminated soil and mining waste were excavated from 820 residences and then capped with clean soil, reducing soil Pb concentrations by 89% (Tirima et al., 2016). Additionally, 2349 children received chelation treatment (Tirima et al., 2016). However, post-remediation monitoring surveys identified work-related Pb exposures and the consumption of crops grown and stored in contaminated areas as ongoing risks in these mining landscapes (Tirima et al., 2017).

**Table 4:** Overview of the potential of bacteria as an amendment to Pb pollution when applied in different experimental conditions within the African context

Sample source	Type of bioremediation agent	Experiment type	Environment	Influence on Pb availability	Reference
Mine waste—Kabwe, Zambia	*Bacterium Oceanobacillus profundus KBZ 3-2*	Laboratory experiment	pH had a direct relationship with O. *profundus* KBZ 3-2 biosorption of Pb	At an initial concentration of 50 mg/L, the highest removal percentage for Pb (II) was 97% through biosorption attributed to chelation-complexation on the present functional groups of EPS excreted by the bacteria)	Mwandira et al. (2020)
Mining area in Abare, Zamfara State, Nigeria	*Pantoea agglomerans*	Spread plate method	The optimum biosorption conditions for Pb were 35 °C and a pH of 7	Metal uptake biosorption percentage revealed that *Pantoea agglomerans* absorbed 99.6% of Pb. Ascribed to possible homeostatic phenomena and the availability of metal-binding sites on the biosorbents	Audu et al. (2020)
Engyiba mining site—Ebonyi State, Nigeria	*Penibacills* sp. Strain SEM1 and *Morganella* sp. Strain WEM7	Laboratory experiment	Bacteria isolates thrived in media containing 5–15% sodium chloride	SEM1 was able to tolerate up to 10,000 mg/L, while WEM7 tolerated more than 10,000 mg/L in both solid and liquid media, and, as such, is a promising tool for the bioremediation of Pb-contaminated water and soil	Orji et al. (2021)
Mining area—Zamfara State, Nigeria	*Aeromonas *sp.*,* Aer*omonas sobria, Alcaligenes faecalis, Bacillus *spp.*, Enterococcus *sp.*, Leptothrix ginsengisoli, Micrococcus *sp.*, Pseudomonas* sp.*, Streptococcus *sp.	Laboratory experiment	pH ranged between 7.1 to 8.2, OC 0.18 to 1.12%, CEC- 1.52 to 3.57 cmol/kg and EC-0.15 to 0.32 ds/m	*Alcaligenes faecalis* strain UBI (MT107249) and *Aeromonas* sp. strain UBI (MT126242) were Gram-negative and are good candidates for genetic modification for bioremediation	Ibrahim et al. (2021)
Mining areas—Anka LGA, Zamfara State (Bagega, Dareta, Abare, and Waramu)	*Bacillus cereus strain BUK_BCH_BTE2*	Incubation experiment	Sucrose and ammonium sulphate at con of 5 g/L and 2.5 g/L aided isolate growth (Co and N fixation) at an optimal temp of 37 °C and pH of 7.0 with an inoculum size of 100 μL throughout 48 h utilizing 1000 mg/L lead nitrate	A concentration of 1000 mg/L Pb (NO_3_)_2_ was found to be the optimum concentration for the isolate, making it suitable for future Pb bioremediation work	Harun et al. (2023)
Farm and mining site in Ebonyi Ste, Nigeria	Rhizobacteria from the rhizosphere of maize plants	Hot plate aqua-regia digestion method	Few studied isolates resulted in P and K solubilization, while others fixed nitrogen	*P. fluorescens* + *B. cereus* maximumly degraded Pb (87.2%) and is recommended for bioremediation of Pb-contaminated soil, while the least (76.0%) was observed in P. fluorescens	Okpara-elom et al. (2024)
Mining site—Anka, Zamfara state Nigeria	*Paenebacillus* sp. strain BUK_BCH_BTE 3 (MT160418) and *Bacillus* sp. strain BUK_BCH_BTE 4 (MT160452)	Laboratory experiment	Optimum growth at 37 °C, a pH of 7.0 over 48 h while utilizing inoculum volumes of 100 μL each and sucrose conc at 10–20 g/L, 5 g/L nitrogen source, and 5 g/L of urea	An optimal Pb concentration of 1000 g/L was obtained by both isolates with up to 3000 mg/L lead nitrate tolerance, hence, locally isolated *Paenebacillus* sp. and *Bacillus* sp. are promising tools for bioremediation of Pb-contamination	Harun et al. (2024)

*Con* concentration, *CEC* cation exchange capacity, *K* potassium, *P* phosphorous, *Na* sodium, *Mg* magnesium, *OC* organic carbon, *Ca* calcium

**Table 5:** Assisted phytoremediation of Pb-polluted soil in mining sites and agricultural fields in African mining landscapes

Location	Concentration (ppm)	Type of soil amendment	Experiment type	physical properties	Influence on Pb availability	Key recommendations	Reference
Gold Mining area—Rafi Local Govt Area in the Nigerian state of Niger	*56*	*Sida acuta* and* Melissa officinalis *L. assisted with *Bacillus safensis* (100 ml/3 weeks) and vermicompost produced from chicken droppings and goat manure	Pot experiment	Both amendments increased soil organic carbon, pH, Na^+^, K^+^, Mg^2+^, and Ca^2+^	After the experiment, both *M. officinalis *L.(5.88 to 12.37 ppm) and *Sida acuta* (6.74–11.8 ppm) removed Pb and reduced soil-Pb concentration	*M.* *officinalis L*. and *S. acuta,* assisted by vermicast and plant growth-promoting bacteria (*Bacillus safensis*), have the potential to remediated metal polluted soils	Sesan et al. (2024)
Zn/Pb and Cu mining-affected wetlands in Kabwe and Copperbelt—Zambia	15 ± 4 to 32,000 ± 5.80	*P. mauritianus* and *Typha* spp.	Naturally grown trees		*P*. *mauritianus* and *Typha* spp. provide the potential for phytostabilization to settle and contain polluted sediments	Need to study other ways to solve the high soil Pb in Kabwe *P*. *Mauritianus and Typha spp. have the potential to phytostabilize metal-contaminated soil sediments*	Nabuyanda et al. (2022)
Cu/Ni Mine tailings—Selebi-Phikwe, Botswana	452.81	Mycorrhizal (– AM and + AM) and fly ash (0% and 10% by wt)	Pot experiment using *Acacia albida*, *Acacia luederitzii,* and *Acacia tortilis* tree species	Fly ash increased the soil pH from From the initial pH 4.3 of the soil to about pH 8.8	Fly ash increased Pb concentration in both the plant root and shoot of *Acacia albida* but reduced it in both *Acacia luederitzii* and *Acacia tortilis,* while mycorrhizal inoculation reduced the Pb concentration in all 3 plant species	*A.* *luederitzii* has potential as a phytoremediation agent in mine tailings, provided that fly ash and mycorrhiza are used during its establishment	Ultra and Manyiwa (2020)

**Table 6:** Organic and inorganic soil amendments for the remediation of Pb-polluted soil in mining sites and agricultural fields in African mining landscapes

Location	Type of soils amendment	Experiment type	Physical properties	Influence on Pb availability	Key recommendations	Reference
Mining area-Kabwe, Zambia	Triple superphosphate (TSP) solution, humate solution and their mixture in a ratio of 1:1	Laboratory experiment	Soil pH was increased using distilled water saturated with calcium carbonate (calcite)	The addition of a humate or phosphate (TSP) solution did not reduce the dissolution of Pb that settled on the plant surface, but reduced plant-Pb bioavailability in soil	-Topsoil removal in cultivated lands -Use of phosphate amendments to treat contaminated soil -Regular monitoring of toxic elements in vegetables cultivated in treated soil	Kříbek et al. (2019)
Farmlands- Kitwe, Zambia	Pumpkin leaves and maize grown in lime (CaCO_3_) (2.0 t/ha^−1^) and chicken manure (5.0 t/ha^−1^)-treated soil	Field experiment	Acidic soil	Concentrations of Pb in pumpkin leaves were above the prescribed FAO/WHO safe limit (0.3 mg kg^−1^) by 33–133%, but lime reduced Pb in pumpkin leaves by 19%. There was no significant interactive effect of lime and manure Pb in the soil	-Consider the spatial environmental variation for effective planning of the application of agronomic amendments in areas with contrasting microenvironments -At lime and manure rates equal or less than 2 t ha^−1^ and 5 t ha^−1^ did not reduce Pb uptake in pumpkin leaves, need for further research	Kaninga et al. (2021)
field-Kabwe-Zambia	Chicken manure (50 t/ha ^−1^), (0.9 t/ha^−1^) triple superphosphate, and a 6 t/ha^−1^ TSP blended fertilizer (BF consisting of NPK fertilizer and composted chicken manure) and maize	Field experiment	Soil was acidic with a pH of 5.7 and CEC of 5.2 cmol + kg^−1^ before the experiment	All soil amendments decreased bioavailable soil Pb concentrations by 29–36%	Chicken manure and phosphate-based soil amendments have significant potential to reduce the concentrations of Pb, Zn, and Cd in maize grown in the study area	Mwilola et al. (2020)
Mining-Ahafo-Kenyasi, Ghana	Gypsum applied at 0, 20, 40, 60, and 80 ton/ha	Field experiment	Gypsum reduced soil organic carbon but increased N, P, and K	Pb in soil was below 3 mg kg^−1^ in all treatments. Pb concentration in cucumber was significantly higher (above 8 mg kg^−1^) during the major rainy season, above the WHO/FAO limits	-There is a need for long-term studies - Need for multi-locational subsoil evaluation with gypsum amendments	Asante et al. (2021)
Mining sites—Kabwe, Zambia	Pigeon-pea biochar pyrolysed at 600 °C in an earth mound kiln applied at 4 wt%	Diffuse gradient in thin film (DGT) experiment	Biochar-induced alkalinity, causing metal hydrolysis and precipitation, but significant sorption also occurred	Biochar treatment significantly (*p* < 0.05) reduced the bioavailable metal Pb by 64 ± 8%, with similar effects detected for metal mobility	-Use of biochar with a higher associated CEC, as both sorption and pH-related mechanisms would contribute to further immobilization of the metals	Brandsvoll, (2021)
Garden sample- Kabwe, Zambia	Chicken manure (CM) and chicken manure-derived biochar (CMB) were made using the pit method and applied at 2 and 4% (w/w)	Greenhouse experiment	CM resulted in the reduction of soil Ph while CMB increased Ph. But Ph was neutral to alkaline in both cases	Bioavailable Pb in the soil decreased with increasing pH between soil pH 6.5 and 7.5, but after pH 7.5, the bioavailable Pb increased as the pH increased. CM and CMB reduced Pb conc in plants. Both CM and CMB did not reduce Pb availability in soil but reduced plant uptake	-Chicken manure and chicken manure-derived biochar application recommended amendments of Pb-contaminated soil due to affordability -Need for further research to justify varying Pb availability post amendment	Mulenga et al. (2023)

*Con* concentration, *CEC* cation exchange capacity, *K* potassium, *P* phosphorus, *Na* sodium, *Mg* magnesium, *Ca* calcium

In Zambia, where significant restoration efforts have also been undertaken, distinct strategies were implemented in 2003 through the Copperbelt Environmental Project (CEP) by Zambia Consolidated Copper Mines Investments Holdings Plc (ZCCM-IH). This marked a significant milestone in addressing environmental and social costs arising from the mining sector. Measures included community sensitization, dredging of the Kabwe canal zone, removal of mine waste from the road dump, removal of topsoil and planting of grass in affected communities, provision of food supplements for children with elevated blood Pb levels, and planting of Moringa oleifera trees for environmental mitigation (The World Bank, 2003; ZCCM-IH Environmental Coordinaton Unit (ZECU), 2002).

Interestingly, the distinct strategies adopted in Nigeria and Zambia yielded varying results. In Nigeria, adaptations of US Superfund soil removal protocols, tailored to local resources, work culture and cultural norms, focused on behavioural modifications and in situ management, rather than contaminant removal, as was done in Zambia (Tirima et al., 2015). These strategies led to a decline in mean blood Pb levels (BLLs) among children in Nigeria from 173 µg/dL to < 20 µg/dL over 4 years. In contrast, in Zambia, mean BLLs remained above 65 µg/dL, with soil Pb levels exceeding 1500 mg kg⁻^1^, thus continuing to endanger successive generations (Tirima et al., 2015). This highlights the importance of adapting strategies to local contexts and incorporating cultural practices for successful remediation. Continuous monitoring and evaluation are vital for refining strategies and ensuring long-term efficacy.

### Bioremediation of Pb-polluted soils in mining landscapes

Long-term adaptation and remediation strategies for Pb-polluted landscapes include biocementation, bioremediation, and phytoremediation, often coupled with the application of organic and inorganic additives to reduce environmental-human Pb transfer. Based on the literature reviewed, the earliest bioremediation activities in Pb-contaminated soil were conducted in 2020 and are limited to West and Southern Africa (Table 4). Interestingly, all reviewed studies utilized local isolates and optimized their environments to achieve optimal growth and Pb removal. In Zambia, *Bacterium oceano bacillus profundus KBZ 3-2* was reported as optimal for Pb removal in the Kabwe Pb-contaminated soils (Mwandira et al., 2020). In Nigeria, *Pantoea agglomerans*, *Penibacills* sp. Strain SEM1 and *Morganella* sp. Strain, *Alcaligenes faecalis* strain UBI (MT107249) and *Aeromonas* sp. strain UBI (MT126242), *Bacillus cereus strain BUK_BCH_BTE2, P. fluorescens* + *B. cereus Paenebacillus* sp., and *Bacillus* sp., were all found to be promising Pb bioremediaters, particularly in neutral pH soil (Audu et al., 2020). This review clearly indicates that different bacteria require specific environmental conditions, such as a neutral pH for effective bioremediation (Table 4).

### Phytoremediation of Pb-polluted soils in mining landscapes

Efforts in phytoremediation for Pb-contaminated mining landscapes have emerged mainly over the past 5 years. However, over two decades ago, Leteinturier et al. (2001) highlighted phytostabilization as an effective remediation approach for Pb-affected Zambian soils, using plant species such as *Indigofera spicata*, *Melinis repens, Cynodon dactylon, Aristida adscensionis,* and *Pennisetum setaceum*. Similarly, Nabuyanda et al. (2022) recommended using naturally occurring species such as *P. mauritianus* and *Typha* spp. for the phytostabilization of Pb-laden sediments by settling and containing polluted sediments (Table 5). Over the years, phytoremediation has advanced to include various organic and inorganic amendments that enhance the efficient remediation of metal-contaminated soils. In Nigeria, a pot experiment involving *Sida acuta* and *Melissa officinalis* L., alongside plant growth-promoting bacteria (*Bacillus safensis*) and vermicompost derived from chicken and goat manure, showed a decrease in Pb availability across all treated soils (Sesan et al., 2024). Lower metal bioavailability was observed in some experimental months with increased soil pH, clay content, or organic matter, whereas in others, low pH and root exudate secretion enhanced metal availability (Sesan et al., 2024). This suggests that interactions between bacteria and vermicompost effectively assisted accumulator plants in phytoextracting Pb from polluted soils. However, cultivating edible crops in such soil could pose risks to human health due to heightened metal availability, especially if hyperaccumulating crops are harvested.

A study in Botswana reported that fly ash increased Pb uptake in *A. albida*. At the same time, the presence of mycorrhizal fungi significantly reduced Pb uptake in shoots by 61% and in roots by 63% compared to uninoculated plants (Ultra & Manyiwa, 2020). In *A. luederitzii*, the combination of fly ash and mycorrhiza resulted in a 69% reduction in Pb uptake in roots and 37% in shoots. However, fly ash application decreased Pb concentration in both shoots and roots of *A. tortilis*, regardless of mycorrhizal inoculation (Ultra & Manyiwa, 2020: Table 5). Therefore, the effects of mycorrhizal fungi and fly ash on Pb uptake vary by plant species and require further optimization for use as amendments in edible crops. Nevertheless, *A. luederitzii* is a promising phytoremediator if both fly ash and mycorrhiza are utilized in its establishment. Similarly, Asante et al. (2021) conducted a related study in Ghana using gypsum but observed no significant reduction in plant Pb uptake across treatments where soil Pb levels were below 2 mg kg^−1^. Notably, Pb concentration in the test crop (cucumber) exceeded the WHO/FAO recommended levels, thus highlighting the need for further optimization of gypsum use as an amendment in Pb-contaminated soils.

### Soil amendments used in the remediation of Pb-polluted soils in mining landscapes

The use of phosphorus-based amendments has shown positive results when applied to Pb-contaminated soils (Kříbek et al., 2019; Mwilola et al., 2020). Kříbek et al. (2019) used triple superphosphate (TSP) solution, humate solution, and their mixture to amend Pb-contaminated soils because phosphate as an amendment can significantly limit Pb bioavailability due to the limited solubility of Pb-phosphates, such as pyromorphite (Table 6). Metal cations can enter phosphate minerals via adsorption, direct ion substitution, coupled substitution within the apatite structure, and the development of discrete trace-metal phosphate phases distributed within the apatite lattice (Kříbek et al., 2019). The study concluded that triple superphosphate can significantly reduce Pb availability in soil (Kříbek et al., 2019) but further optimization over more extended periods and under different environmental stresses is needed. In a similar study, Mwilola et al. (2020) observed that chicken manure, triple super phosphate, and a blend of fertilizer and composted chicken manure reduced bioavailable Pb in soil by 30%, 36%, and 29% in maize fields, respectively (Table 6). However, Pb concentration in maize grain was observed to be above the joint FAO/WHO maximum permissible limit of 0.2 mg kg^−1^ for human health safety (Mwilola et al., 2020). These findings highlight the need to further investigate the potential of phosphorus-based amendments in the remediation of Pb-contaminated soils.

Similarly, lime and chicken manure have been studied as amendments for Pb-contaminated soil in some mining landscapes. Sichilima et al. (2024) observed that lime stabilized Pb in soils through a pozzolanic reaction and concluded that lime, in combination with steel slag (unlike char and ash), significantly reduced bioavailable Pb in soil. Kaninga et al. (2021) also observed that applying lime and chicken manure effectively reduced Pb concentration in maize grain, but significant amounts accumulated in the plant stover. Although lime shows potential as a Pb-contaminated soil amendment, challenges such as cost implications need to be taken into consideration (Sichilima et al., 2024), while further optimization for complete above-ground Pb reduction in plants is also warranted (Kaninga et al., 2021).

Recently, scholars have suggested using biochar as an amendment for heavy metal-contaminated soils. Biochar is a carbon-rich substance produced by heating organic materials (e.g. wood, crop residues, or manure) in a low-oxygen environment through pyrolysis (International Biochar Initiative, 2012). It is known for its potential to improve soil quality, retain water, sequester carbon and immobilize heavy metals. Biochar’s efficacy is primarily mediated by its capacity to augment soil pH and Cation Exchange Capacity (CEC), which are key determinants of metal bioavailability. By increasing specific surface area and providing active functional groups, biochar enhances the soil’s adsorptive capacity and alkalinity, effectively transitioning Pb from a mobile to an immobilized state (Albert et al., 2021).

Though limited, studies have been conducted in African mining landscapes using biochar. Yoshii et al. (2020) reported on the remediation of contaminated soils using chicken manure, chicken manure-derived biochar, urea, and lemongrass on Pb-contaminated soil in Kabwe, Zambia. The study found that biochar did not significantly affect exchangeable Pb levels, indicating the need for further studies that also consider its influence on pH and CEC, among other variables (Yoshii et al., 2020). In a study conducted by Mulenga (2023), chicken manure-derived biochar increased soil pH to a peak of 8.27 ± 0.14, which was attributed to the presence of free basic cations in the biochar. However, there was an increase in available Pb in the soil compared to the control, ascribed to cation competition on biochar adsorption sites (Mulenga et al., 2023). In contrast to the aforementioned studies, Brandsvoll (2021) used pigeon pea biochar and observed a significant decrease in lead availability and mobility, attributing this to increased soil pH and organic matter (Table 6).

In West Africa, the use of biochar, though equally limited, has been explored as an amendment for Pb polluted from various sources. A study conducted with soil samples collected from various Pb smelting slag-contaminated soils in Ibaden, Nigeria, used Rice husk (RH) and Cashew nut shell (CNS) biochars and compost-modified biochars in comparison to compost for stabilizing Pb in lead smelting slag (LSS)-contaminated soil (Ogundiran et al., 2015). The study found that rich husk-derived biochar increased soil pH from acidic to alkaline, reduced CaCl_2_-extractable Pb in the soil, reduced plant uptake, and improved overall plant performance (Ogundiran et al., 2015). Biochar studies have also been conducted on soil samples collected from the surroundings of botanical gardens at the University of Ilorin in Kwara State, spiked with used spent engine oil (Ogunremi et al., 2025). Standard biochar was reported to reduce plant Pb availability attributed to the increase in soil pH, while sorghum-derived biochar enhanced the biomass yield of *t-diversifoliar* (Ogunremi et al., 2025). Other studies using biochar have been conducted in Nigeria, but with a focus on other plant chemical properties and plant growth parameters that may influence Pb availability in plant tissues when applied to Pb-contaminated soils (Ayo et al., 2025; Musibau Oyeleke Azeez et al., 2025).

These observed discrepancies are, to some extent, linked to the complexity of biochar, as its effectiveness depends on factors such as feedstock type, pyrolysis conditions, application rates, and production methods (Haddad & Lemanowicz, 2021; Jacob et al., 2022; Uchimiya et al., 2011). This complexity has resulted in a lack of well-characterized biochar produced under standardized conditions. Consequently, the varied and sometimes conflicting effects of biochar on Pb bioavailability in soil and plant responses cannot be compared. Therefore, optimizing biochar is essential given its complexity and the varying results observed in Zambia. This review also noted the potential of phosphorus-based amendments when applied to Pb-contaminated soils (Kříbek et al., 2019; J. Mandal et al., 2022; Mwilola et al., 2020), suggesting that exploring synergies with phosphorus-based amendments may enhance biochar’s potential as an amendment for Pb-contaminated soils.

## Conclusion and future perspectives

This review highlights that monitoring of lead (Pb) contamination has predominantly been conducted in Southern and Western Africa, while a notable gap exists in Northern and Eastern Africa. These regions are most adversely affected by Pb pollution, with soil concentrations often exceeding permissible limits. Furthermore, assessments of Pb pollution have primarily focused on blood lead levels (BLLs), while the use of alternative biomarkers has been limited. Pb exposure is mainly linked to direct ingestion and inhalation of Pb-contaminated dust particles, but the extent of community exposure through the food chain remains a significant concern. The entry of Pb into the food chain largely results from dust deposition and bioaccumulation, which poses a serious threat to human health. This review indicates that immediate interventions in Pb-affected communities often have a limited lifespan due to funding constraints. Similarly, bioremediation efforts have been largely confined to lab and greenhouse settings, which limits understanding of their scalability and underscores the need to consider the interplay among technical performance, site-specific soil requirements, social acceptability, and the practical capacity for community-led initiatives. While traditional remediation approaches have frequently relied on expensive physical interventions, Nature-based solutions—such as soil amendments, microbial enhancers, and assisted phytoremediation—show promise. Among these, biochar stands out as the most effective and sustainable amendment due to its high surface area, pH balance, porosity, and functional groups that enhance Pb immobilization and improve soil physicochemical properties. Additionally, biochar fosters microbial activity, supports plant growth, and offers long-term stability in soils, thereby reducing Pb bioavailability, limiting crop uptake, and mitigating risks to food security and public health. Nonetheless, the potential of biochar as an amendment for Pb-contaminated agricultural soils remains underexplored, primarily due to the absence of contextualized guidelines for biochar production and a systematic analysis of its properties, characteristics, and utilization directives.

### Future perspectives

Future perspectives for the management and control of environmental Pb pollution and remediation strategies in African mining landscapes are summarized as follows:

1. Implement sensitization programs that will educate communities on Pb pollution pathways and protective measures.
2. Future studies must seek to address the monitoring gap in soil Pb contamination in Northern and East African mining landscapes.
3. Future studies must be conducted on strategies for regular monitoring of heavy metals, both in the atmosphere and in agrarian soils and regular monitoring of Pb content in crops grown in mining landscapes.
4. There is a need to conduct studies that will use biomarkers other than BLL, highlight the actual illnesses recorded in African mining areas as a direct result of Pb mining and justify its correlation with poor food nutrition.
5. There is a need to explore large-scale studies that integrate socially acceptable practices that can easily be adopted and replicated by affected communities to increase acceptability and ease of implementation.
6. There is need for studies that will contribute to the formulation of site-specific frameworks for biochar application in Pb-contaminated agricultural soils and effectively mitigat its translocation into the food web.

## Data Availability

The authors declare that the data supporting the findings of this study are available within the paper. Additional data and information can be sourced from the cited references and online databases or sources.
